# Bayesian inference of metabolic kinetics from genome-scale multiomics data

**DOI:** 10.1371/journal.pcbi.1007424

**Published:** 2019-11-04

**Authors:** Peter C. St. John, Jonathan Strutz, Linda J. Broadbelt, Keith E. J. Tyo, Yannick J. Bomble

**Affiliations:** 1 Biosciences Center, National Renewable Energy Laboratory, Golden, Colorado, United States of America; 2 Department of Chemical and Biological Engineering, Northwestern University, Evanston, Illinois, United States of America; The Pennsylvania State University, UNITED STATES

## Abstract

Modern biological tools generate a wealth of data on metabolite and protein concentrations that can be used to help inform new strain designs. However, learning from these data to predict how a cell will respond to genetic changes, a key need for engineering, remains challenging. A promising technique for leveraging omics measurements in metabolic modeling involves the construction of kinetic descriptions of the enzymatic reactions that occur within a cell. Parameterizing these models from biological data can be computationally difficult, since methods must also quantify the uncertainty in model parameters resulting from the observed data. While the field of Bayesian inference offers a wide range of methods for efficiently estimating distributions in parameter uncertainty, such techniques are poorly suited to traditional kinetic models due to their complex rate laws and resulting nonlinear dynamics. In this paper, we employ linear-logarithmic kinetics to simplify the calculation of steady-state flux distributions and enable efficient sampling and inference methods. We demonstrate that detailed information on the posterior distribution of parameters can be obtained efficiently at a variety of problem scales, including nearly genome-scale kinetic models trained on multiomics datasets. These results allow modern Bayesian machine learning tools to be leveraged in understanding biological data and in developing new, efficient strain designs.

## Author Summary

Genetic engineering of microbes is a promising strategy to enable more efficient and environmentally friendly production routes for chemicals and materials traditionally produced from petroleum. While the tools to both edit microbial genomes and measure the resulting changes to cellular physiology are growing increasingly efficient, our ability to predict which genetic interventions will have the greatest likelihood of achieving the desired phenotype is still lacking. In particular, computational methods are needed that are able to efficiently ingest the increasing amount of data generated from large biological datasets to guide subsequent rounds of experiments. This study presents an efficient algorithm coupled to modern computational tools that permits a closer integration of modeling in the design, build, test, and learn cycle of genetic engineering.

## Introduction

Microbial metabolism offers a promising route for the sustainable production of fuels and chemicals. Fine-tuning enzyme expression to optimize fluxes within these metabolic networks for maximum titer, rate, and yield is a critical step in improving bioprocess economics [1]. Towards this goal, the process of metabolic engineering has been formalized into an iterative Design—Build—Test—Learn cycle that leverages new improvements in the efficiency of strain construction and characterization techniques [2]. Metabolic engineers now have a greater ability to fine-tune gene expression of both native and exogenous pathways and construct new strains in rapid succession. Characterization of the resulting strains has also become increasingly detailed with the growing availability of transcriptomic, proteomic, and metabolomic analysis techniques [3]. These methods, collectively termed multiomics, measure relative changes in gene, protein, or metabolite concentrations across different strains or growth conditions [4]. While the capabilities of the build and test stages have grown considerably in recent years, utilizing multiomics data to make informed decisions about future strain improvements remains a major challenge in modern bioengineering [5, 6].

Central to this question is determining how steady-state metabolic fluxes are controlled. These fluxes arise through a vast network of enzymes with complex regulation and nonlinear kinetics. Constraint-based models offer one strategy for predicting metabolic fluxes by investigating feasible steady-state phenomena by placing constraints on reaction fluxes in accordance with stoichiometric [7], thermodynamic [8], enzymatic [9], and regulatory (reviewed in [10]) rules. However, some predictions require an explicit consideration of reaction kinetics that are not considered in constraint-based modeling. For instance, the rate-limiting enzyme in a multi-step pathway cannot be determined without knowing the kinetics of each enzyme and the concentration of each metabolite [11].

Enzymatic rate rules involve a number of (typically unknown) parameters: variables which must be determined from experimental data. While *in vitro* experiments have characterized the most common enzymes, the vast majority of enzymes lack explicit kinetic information [12]. Alternatively, parameters for kinetic models can be fit by using phenotypic measurements of how metabolic fluxes change following perturbations to enzyme expression or growth media. An important consideration in this approach is the treatment of uncertainty, since many sets of kinetic parameters might reproduce the same observed steady-state phenotypes. Metabolic ensemble modeling (MEM) achieves this result by repeatedly sampling kinetic parameters from within feasible bounds, keeping only those parameter sets that match observed phenotypes to within some tolerance [13–16]. In a somewhat similar manner, the ORACLE framework generates sets of possible parameters using a Monte Carlo approach, keeping only those that match experimental measurements [17–20]. For ensemble modeling this selection technique has more recently been formalized as Bayesian inference and solved via approximate Bayesian computation, in which parameter values are drawn from prior distributions of feasible values, simulated phenotypes are compared against experimental observations, and the resulting posterior distributions are updated [21].

A major limitation of ensemble-based modeling has been its ability to scale both to larger datasets as well as larger kinetic models, since larger datasets cause a higher fraction of samples to be discarded and larger kinetic models increase the computational cost of each simulation. Computation times on the order of hours per parameter sample have been encountered even for relatively small systems [13, 21], and despite improvements to the computational efficiency of MEM [22, 23] the method remains limited to both small models and a few experimental observations.

In this study, we develop a scalable analog of metabolic ensemble modeling, capable of inferring posterior distributions in kinetic parameters of large metabolic models with multiomics-sized datasets. We sidestep many of the previously discussed computational bottlenecks through the use of linear-logarithmic (linlog) kinetics as an approximate reaction rate rule [24, 25]. Linlog kinetics have a close connection to metabolic control analysis (MCA), which links effects of local perturbations (*i.e.*, changes to enzyme expression) to changes in the resulting steady-state concentrations and fluxes [26, 27]. Most critically, the use of linlog kinetics greatly simplifies the simulation of steady-state fluxes and metabolite concentrations and allows the use of modern and efficient Bayesian inference algorithms [28, 29]. We show that this method is capable of providing systems-level insight into metabolic kinetics for a wide range of kinetic models and dataset sizes. First, we demonstrate the method on a simple *in vitro* example, showing that the method is flexible enough to capture allosteric interactions between metabolites and enzymes. We next show that the method appropriately captures uncertainty in estimated parameters, revealing significant flux control coefficients (FCCs) for only the most likely enzyme perturbations in the case of limited biological data. Finally, we employ the method to integrate thousands of individual metabolomic, proteomic, and fluxomic data-points with a large-scale model of yeast metabolism. We therefore show that the field of metabolic modeling can take full advantage of recent advances in the fields of probabilistic programming, machine learning, and computational statistics, and that ensemble-based approximate kinetic modeling approaches are capable of scaling to genome-sized models and datasets to provide interpretable and actionable insight for strain engineers.

## Results

### Bayesian inference and linlog kinetics correctly predict allostery in an *in vitro* linear pathway

**Fig 1:**
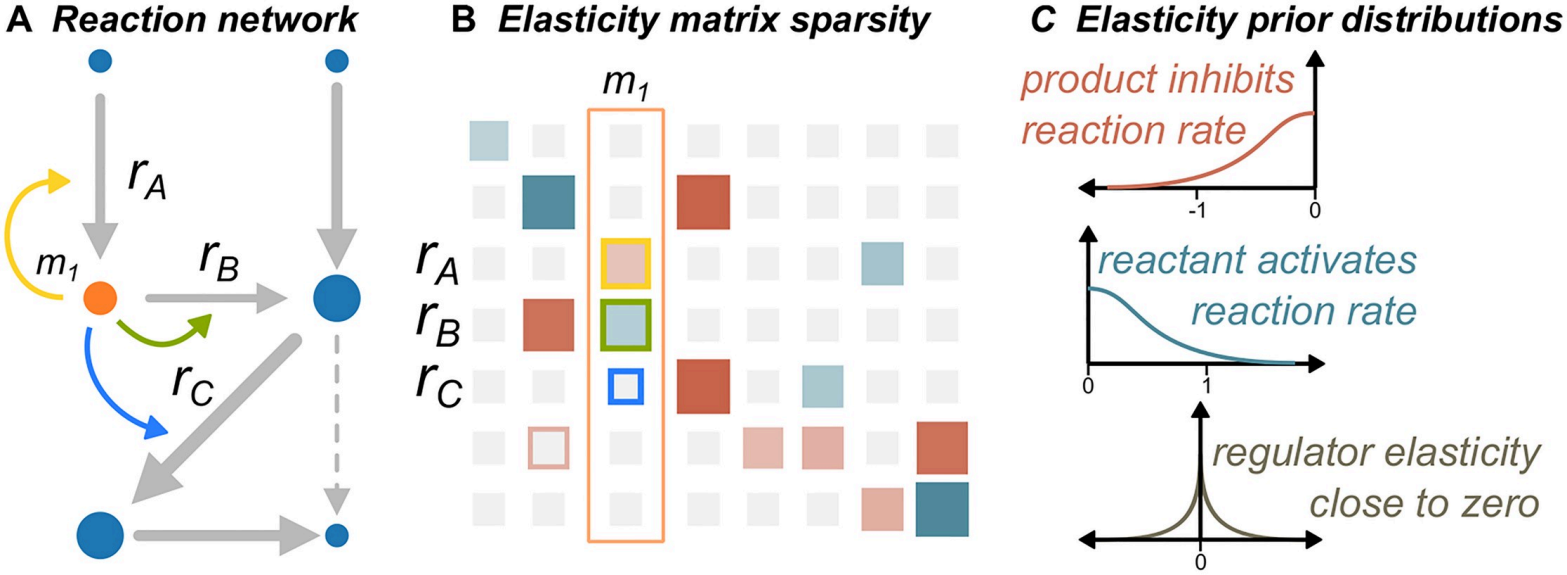
Overview of the modeling framework. (A) The stoichiometry of the reaction network is used to determine prior distributions for the elasticity parameters. (B) The elasticity parameters represented in matrix form, where the column outlined in orange corresponds to *m*_1_ in (A). (C) For a metabolite *m*_1_, the prior distribution has predominately negative support for reactions in which the metabolite is a product (*r*_*A*_), positive support for reactions in which the metabolite is a reactant (*r*_*B*_), and a zero-centered, sparsity inducing prior for reactions in which the metabolite does not participate (*r*_*C*_).

The kinetic models we consider in this study are parameterized with a matrix of elasticity values, where rows correspond to enzymes and columns correspond to metabolites. Values in this matrix reflect how an increase in a given metabolite’s concentration will affect the reaction rate of the corresponding enzyme. Elasticities are unitless parameters defined as the normalized derivative of reaction rate with respect to metabolite concentration. Positive elasticities therefore indicate that higher metabolite concentrations will increase reaction rate, while negative elasticities indicate that higher metabolite concentrations will decrease reaction rates. For a reactant, a small yet positive elasticity implies the enzyme is saturated and further increases in metabolite concentration will not effectively increase the rate. A substantial negative elasticity associated with a reaction product might indicate a reaction close to equilibrium, where changes to product concentration are significant in changing the forward rate. Since the elasticities are represented by a dense matrix, other regulatory interactions including substrate-level inhibition or activation can be captured through non-zero elasticity values associated with the enzyme-regulator pair. We therefore seek to find distributions in the values of this elasticity matrix that are consistent with experimentally observed metabolite, flux, and enzyme measurements. While this approach allows the possibility that all metabolites could affect all reaction rates, we constrain metabolites not participating in a reaction to have a very low probability of affecting flux while still allowing for a small chance of allosteric regulation. An overview of the methodology is presented in Fig 1, and explained in greater detail in the Methods section.

**Fig 2:**
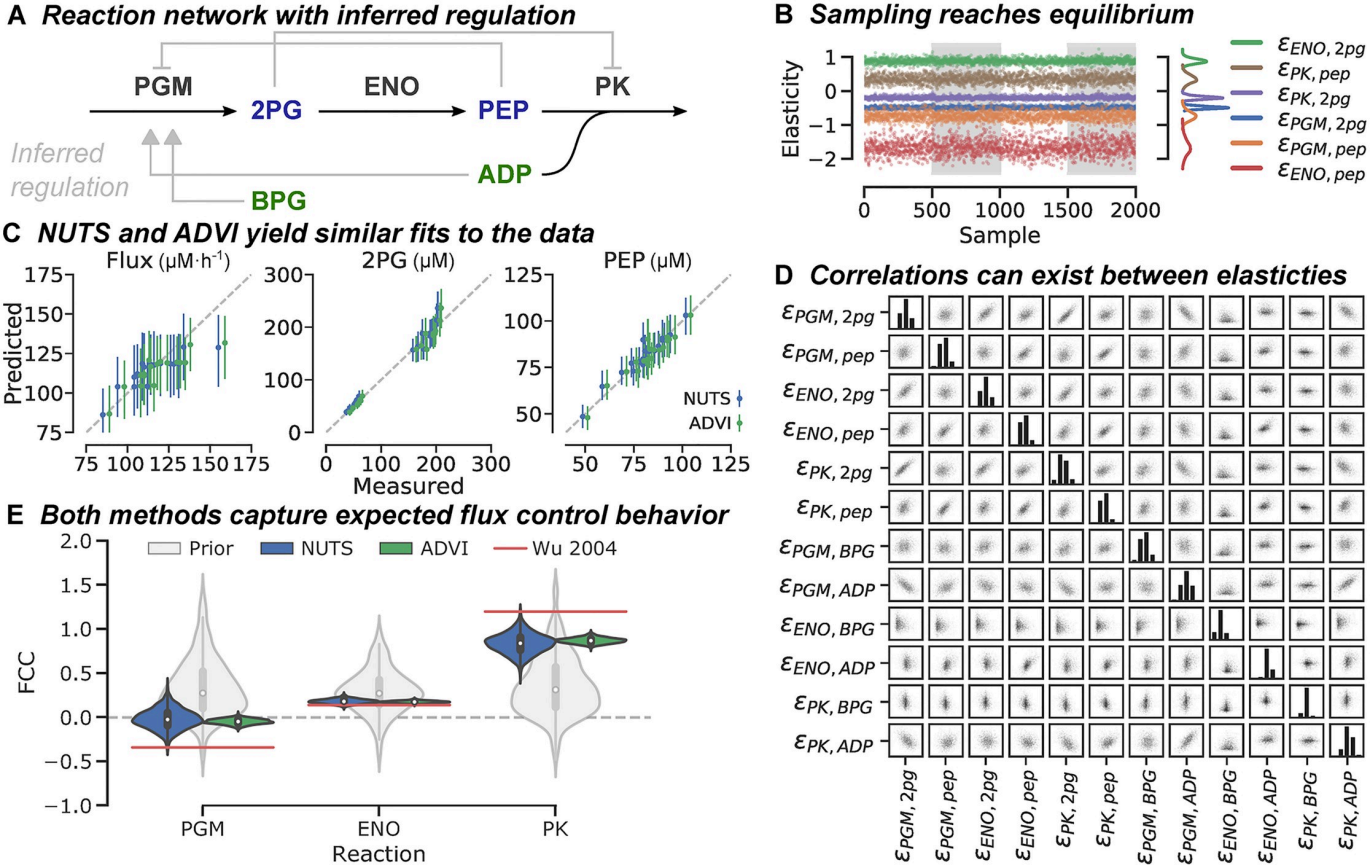
*In vitro* pathway inference. (A) Schematic of the considered pathway. Inferred allosteric interactions are shown in gray, in which arrows indicate an activation, while bar-headed lines indicate inhibition. (B) Traces for $\epsilon_{x}^{*}$ values as estimated by NUTS. Samples come from four parallel chains stacked together as indicated by the shaded regions. Resulting posterior densities are indicated by the inset on the right. (C) Posterior predictive distributions of steady-state flux and metabolite concentrations. Points represent medians of the posterior predictive distributions, with lines extending to cover the 95% highest posterior density. Slight jitter was added to differentiate the distributions as estimated by NUTS and ADVI. (D) Pairplot of the posterior distributions of elasticity variables as estimated via NUTS. Strong correlations can exist between fitted parameters, which are missed by the mean-field ADVI approximation. (E) Violin plot of distributions in FCCs as estimated by the two inference methods. Median and inner quartile range are indicated by the inner box plots, overlaid on a kernel density plot of each distribution.

While the primary purpose of the proposed modeling framework is to parameterize genome-scale kinetic models from large, multiomics datasets, we first demonstrate the method on a simple linear pathway. We re-fit a simple three-reaction model [30] to steady-state *in vitro* flux and concentration data for a reconstructed subsection of lower glycolysis [31]. A schematic of the considered pathway is shown in Fig 2A. The model consists of two internal metabolite species, 2-phosphoglycerate (2PG) and phosphoenolpyruvate (PEP), and two metabolites with externally-controllable concentrations, adenosine diphosphate (ADP) and 2,3-bisphosphoglycerate (BPG). The model consists of three reactions in series, phosphoglycerate mutase (PGM), enolase (ENO), and pyruvate kinase (PK); therefore each carries the same flux at steady-state. The dataset consists of 19 separate experiments, each of which specifies the enzyme loadings and external metabolite concentrations together with the resulting internal metabolite concentrations and steady-state flux.

Bayesian inference is a technique that uses Bayes’ theorem to construct probability distributions in model parameters from experimental data. Inputs to Bayesian inference include both a likelihood model, which describes the probability of the observed data occurring as a function of model parameters, and a prior distribution that encompasses all values that model parameters are likely to take before the experimental data is considered. Numerical techniques then invert these functions to find the posterior distribution: the conditional probability of the parameter after observing the experimental data. The choice of prior distributions can also be used for regularized regression. In this study, Laplace priors (with a sharp peak near zero) are placed on elasticities for metabolite-reaction pairs in which the metabolite does not directly participate, resulting in a posterior elasticity close to zero unless sufficient evidence is present to indicate activation or repression.

The likelihood model in this example assumed the experimental errors were normally distributed, and prior distributions ensured that estimated enzyme elasticities were reasonable. Specifically, we assumed elasticities associated with reactants to be predominately positive, those associated with products to be predominately negative, and those for metabolites not participating in a given reaction to be close to zero (Fig 1C). More details on the specific prior distributions chosen are given in the Methods.

Computational methods for Bayesian inference typically fall into two broad categories. Markov chain Monte Carlo (MCMC) methods exactly sample the posterior distribution by constructing a stochastic process that ultimately converges to a stationary distribution, representing the true posterior. However, convergence of these methods is not guaranteed, and high dimensional posteriors (*i.e.*, lots of parameters) can be numerically difficult to sample. As an alternative, variational Bayesian methods approximate the true posterior with an analytically tractable distribution (for example, a Gaussian), and minimize the dissimilarity between the true and approximate posterior through parameter optimization. While the estimated posterior is only an approximation of the true distribution, variational approaches have been shown to be capable of scaling to neural-network sized models with millions of parameters [32].

In this study, two inference algorithms were used to numerically estimate posterior distributions in elasticity parameters. First, a type of MCMC algorithm called the No-U-Turn sampler [28] (NUTS), and second, an implementation of variational inference known as automatic differentiation variational inference (ADVI) [29]. Four independent sampling chains for the NUTS sampler converged to similar posterior distributions in elasticity values (Fig 2B), taking under 10 minutes on a personal computer, while the stochastic gradient descent optimizer of ADVI converged to a small objective score after 25,000 iterations in less than 40 seconds (S4 Fig). Comparing the results of the two inference methods indicates that both methods yield similar conclusions. A posterior predictive check for both inference methods indicates that the measured experimental data is well-captured by the model (Fig 2C). Both inference algorithms correctly predict allosteric regulation. Despite predicting off-target regulation would be minor, all elasticity values in the internal metabolite elasticity matrix, $\epsilon_{x}^{*}$, were confidently nonzero. Inferred regulatory interactions, which were all consistent between both inference methods, are shown in gray in Fig 2A. These include a strong repression of PGM by PEP, and a weaker repression of PK by 2PG. These off-target regulatory interactions (with similar elasticity values) were also found through the original linear regression approach of Wu *et al*., 2004. For the external metabolite species, only one of the four possible off-target regulatory interactions, ADP activation of PGM, resulted in a posterior distribution that was confidently nonzero. This relatively weak interaction was rejected by the original linear regression method through a combination of experimental and mathematical reasoning, but underscores that interactions between metabolites and fluxes are inherently difficult to predict from this type of data: direct vs. indirect interactions often look similar, and causality is often impossible to establish. Notably, the posterior distribution as estimated via NUTS contains a rich amount of information on the identifiability of elasticity values (Fig 2D). Strong correlations in estimated parameters can be seen where the two elasticities share either a metabolite or reaction. While this phenomena is common where models show structural identifiability [33], these correlations are not captured by the approximate ADVI method, which fits independent Gaussian distributions for each variable.

Once appropriate parameter distributions that fit the data have been found, we can determine which enzymes in the pathway are rate-limiting by using the posterior predictive distributions in flux control coefficients (FCCs). In this example, since steady-state fluxes are constrained to be equal for all three reactions, the FCCs are a vector of three coefficients that determine whether increasing enzyme concentration will increase or decrease pathway flux. Fig 2E shows posterior distributions in FCCs as estimated with both inference methods. These are compared against FCC distributions resulting from only the prior distributions on elasticity parameters, without considering any experimental results. Prior distributions (light gray) are similar between all three enzymes and indicate no structural bias on FCCs. The data therefore indicate that pyruvate kinase (PK) is the limiting enzyme at the reference state. We also compare our FCC estimates against those originally calculated via linear regression, assuming specific allosteric interactions between metabolites and enzymes that differ from those found to be significant through our approach. Our estimates of FCCs closely match those found by Wu *et al*., 2004, indicating that systems-level properties are relatively insensitive to the particular parameterization used to capture allosteric regulation.

The close agreement of the estimates provided by the approximate ADVI method to the more accurate NUTS traces in elasticities and FCCs is an important result. As most applications in metabolism involve a larger reaction network, approximate inference methods are likely the only techniques that will scale to biologically-relevant *in vivo* examples. We therefore rely only on ADVI for subsequent examples that deal with larger reaction networks.

### Bayesian inference and linlog kinetics determine optimal enzyme targets from limited data

**Fig 3:**
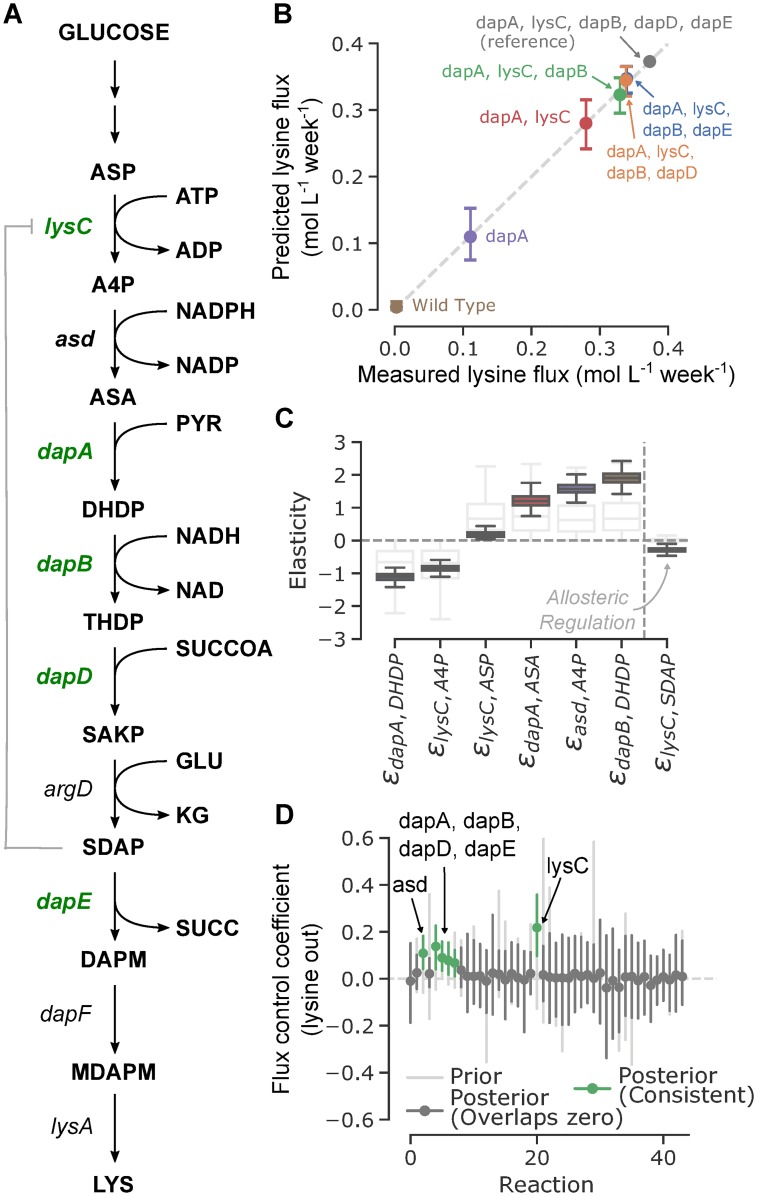
Inference on a medium-scale metabolic network with limited data. (A) Schematic of a portion of the considered metabolic network corresponding to lysine biosynthesis. Reactions shown in green were experimentally determined to improve lysine yields. The allosteric regulation of lysC by SDAP (N-succinyl-L,L-2,6-diaminopimelate) inferred by the model is shown in gray. (B) Experimental vs posterior predictive distributions for lysine flux. Error lines extend to cover the 95% highest posterior density (HPD) interval. (C) Distributions of elasticities informed by the experimental results. Prior distributions for these elasticities are shown in light gray. The one allosteric elasticity confidently inferred is shown as the last entry. (D) Flux control coefficients for each reaction in the model. Prior distributions (light gray) are mostly centered around zero. Posterior distributions (dark gray) are highlighted in green if their 95% HPD does not overlap zero. All lines indicate 95% HPD ranges, dots indicate median.

We next demonstrate how the inference framework can be used to suggest enzyme targets in a many-reaction network that includes branching and shared co-factors. The problem we consider was previously examined through ensemble metabolic modeling [34], and involves predicting which manipulations might further increase lysine production in engineered *E. coli* strains. The experimental data consists of lysine flux measurements (in mol L^−1^ week^−1^) from six sequential enzyme overexpression experiments, all of which were observed to improve L-lysine yields [35]. The metabolic model used for inference comprises 44 reactions and 44 metabolites covering central carbon metabolism and lysine production, taken from Contador *et al*., 2009. A schematic of the reaction network is shown in Fig 3A.

Posterior distributions in the elasticity matrix were estimated using ADVI as with the *in vitro* example, with the optimization taking under three minutes (as compared with days for a comparable ensemble-modeling based approach [22]). The posterior predictive distribution for each strain closely matches the measured lysine fluxes [35], indicating the model is capable of reproducing experimental behavior (Fig 3B). Of the 133 ‘kinetic’ elasticity terms, those corresponding to reactions in which a metabolite directly participates, only six were substantially constrained by the experimental data. Posterior distributions for these elasticities are shown in Fig 3C, and the inferred regulatory interaction is shown in gray in Fig 3A. Unsurprisingly, nearly all of these elasticities involve reactions and metabolites in the lysine synthesis pathway, the only portion of the model for which overexpression results were provided.

We next calculated prior and posterior distributions in FCCs. Because only a limited selection of data was available to constrain the elasticity values, only 6 of the 44 reactions had a confidently nonzero FCC. However, five of these reactions were the same as previously specified as successful modifications for improving lysine flux (dapA, lysC, dapB, dapD, dapE) [34]. The remaining reaction, asd (aspartate-semialdehyde dehydrogenase), is a part of the same pathway as the other successful overexpressions. Prior and posterior distributions in FCC values for lysine export are shown in Fig 3D. While previous ensemble modeling results indicated several additional enzyme overexpressions that might increase lysine pathway flux, our analysis demonstrates that the observed sequential overexpression experiments could be recreated through a wide variety of possible parameterizations with a resulting wide distribution in possible flux responses. These results show that the method generalizes well to the case where insufficient data is provided to constrain model predictions and underscores the importance of rigorously characterizing posterior parameter space to determine the full range of possible model responses.

### Informing strain design through multiomics

**Fig 4:**
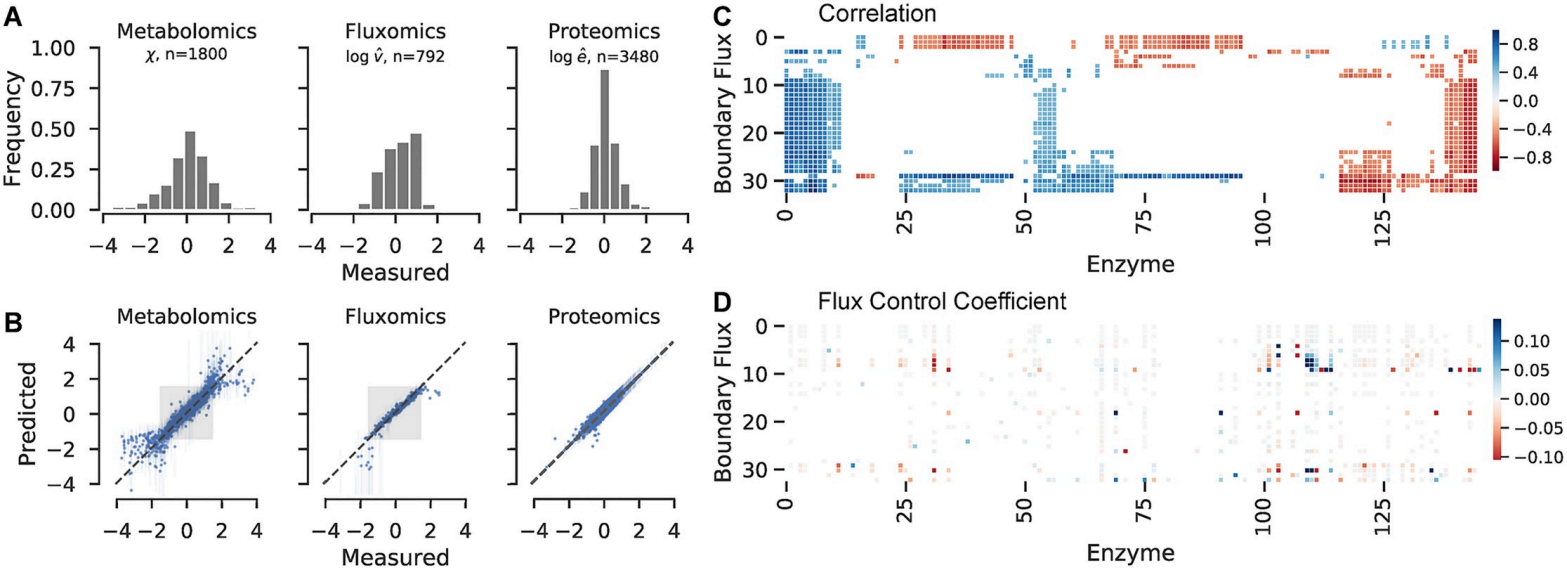
Parameterizing a genome-scale kinetic model with multiomics data. (A) Distributions in log-transformed (unitless) experimental data after normalizing with respect to the phosphate-limited reference state. (B) Posterior predictive distributions after fitting with ADVI. Higher weight was given to experimental datapoints close to the reference state (±1.5) as indicated by the gray boxes. (C) Heat map of correlation coefficient between experimental enzyme measurements (x-axis) and experimental boundary flux measurements. Boundary fluxes and enzymes are sorted with hierarchical clustering. (D) Heat map of FCC as estimated from posterior parameter distributions. Boundary flux and enzyme ordering match those determined in (C). Colors represent medians of the posterior predictive distributions, FCCs with a direction that could not be confidently determined are colored white.

The main strength of the proposed method is its ability to constrain kinetic parameters using multiomics data, even for large-scale metabolic systems. We therefore demonstrate the method using literature data on metabolomics, proteomics, and quantification of exchange fluxes for 25 different chemostat experiments with yeast [4]. The dataset comprises 5 different media conditions, each of which was run at 5 different dilution rates. We adapt a large-scale metabolic model of yeast metabolism that includes many of the genes, metabolites and boundary fluxes of interest [36]. The adapted model contains 203 metabolites and 240 reactions and was obtained by removing blocked metabolites and reactions under growth on glucose. In total, the experimental data consists of 1800 metabolite measurements, 792 boundary flux measurements, and 3480 enzyme measurements (omitting the chosen reference state), shown in Fig 4A. Since the linlog inference framework only uses relative changes to enzyme, flux, and metabolite concentrations with respect to a reference state, it can naturally ingest large-scale multiomics datasets without the need for absolute quantification.

In fitting the observed steady-state phenotypes, the model has to account for not only experimental error in measured enzyme concentrations, but also for potential changes in gene expression in unmeasured enzymes. We therefore place prior distributions on log enzyme concentrations for each condition that drive enzyme changes towards their measured values, or, if the reaction is not measured, towards zero (unchanged). Thus, we allow unmeasured enzyme concentrations to deviate from zero only if there is sufficient evidence. The model parameters therefore include 915 elasticities associated with direct kinetic interactions, 23,684 elasticities associated with potential off-target allosteric regulation, 5,760 enzyme expression levels (240 enzymes over 24 experiments), and 192 external metabolite concentrations (8 metabolites over 24 experiments), for a total of 30,551 parameters. While this number is far greater than the number of experimental data points, regularization forces roughly 85% of these parameters to be zero.

The model was fit using ADVI, with the 40,000 iterations completing in approximately 5 hours on a single compute node. Posterior distributions in parameter values indicate that the model is able to fit the observed experimental data while using relatively few of the additional regulatory parameters. Of the 23,684 regulatory elasticities, only 153 (0.65%) were confidently nonzero. However, we note that determining mechanistically accurate regulatory interactions from observations of steady-state flux behavior is inherently difficult. For instance, for a regulatory pathway in which A regulates B and B regulates C, identifiability issues might cause the pathway to be modeled as A regulates B and A directly regulates C. While poorly identifiable, the impacts of these alternative regulatory topologies on FCCs are largely similar. Of the 50 unmeasured enzymes, only half were nonzero in at least one experimental condition, and overall only 35% of the available unmeasured enzyme expressions differed from their reference state value. Since reaction flux can be controlled through both changes in enzyme expression and metabolite concentrations, these results indicate that the model is overly relying on changes in enzyme expression to capture the observed metabolic phenotypes.

### Bayesian inference and linlog kinetics better select engineering targets than a non-structured correlation analysis

A common goal in strain engineering is to find gene targets for increasing the yield of a given metabolic product. We therefore look at relationships between enzymes with measured protein concentrations and measured boundary fluxes as a way to identify putative targets. In a traditional statistical approach, correlations between enzyme levels and metabolite fluxes might be used to further enhance production of a desired metabolite. Fig 4C shows a heat map of Pearson correlation coefficients between enzyme expression levels (as determined through proteomics) and measured metabolite boundary fluxes. A permutation test was performed to determine correlations significant at the *α* = 0.05 confidence level; non-significant correlations were masked from the array. In this map, hierarchical clustering is used to reveal clear groups of metabolites and enzymes that vary together in the experimental data. A larger version of this image, with labelled axes, is shown in S9 Fig. However, correlations between proteins and metabolite boundary fluxes do not necessarily imply that a particular enzyme is involved in directing flux to a particular product. For instance, several of the highest correlations exist between methionine synthase and the relatively distant amino acid products alanine, arginine, and histidine. The top ten enzyme-boundary flux correlations are shown in Table 1.

**Table 1:** Largest significant correlations between measured enzymes and measured boundary fluxes.

Enzyme	Boundary	*ρ*
Methionine synthase	L-Alanine	0.910
Glycine hydroxymethyltransferase, reversible	L-Alanine	0.884
Glycine hydroxymethyltransferase, reversible	Pyruvate	0.866
3’,5’-bisphosphate nucleotidase	Succinate	0.854
Methionine synthase	L-Arginine	0.850
Argininosuccinate lyase	Succinate	0.844
Phosphoserine transaminase	Succinate	0.844
Asparagine synthase (glutamine-hydrolysing)	Succinate	0.839
Methionine synthase	L-Histidine	0.835
Imidazole-glycerol-3-phosphate synthase	Succinate	0.835

We compared this traditional statistical correlation approach to our framework. FCCs that are estimated through Bayesian inference and linlog kinetics offer an alternative approach for determining potential enzyme targets that more systematically considers the effects of metabolic connectivity, stoichiometry, and kinetics compared to the correlation analysis which views the system as a black box. Before considering posterior distributions in FCCs, we first look at whether the prior assumptions on enzyme elasticities and model stoichiometry result in any confidently nonzero values. From the prior predictive distribution, only 6 enzyme-boundary flux pairs have a significantly nonzero FCC, and typically involve reactions directly associated with metabolite production. For instance, a positive FCC is associated with asparagine synthase and valine transaminase on asparagine and valine export, respectively. A heat map of FCCs calculated from the fitted posterior elasticity matrix is shown in Fig 4D, in which FCC distributions that overlap are colored white. Unlike the map of correlation coefficients, FCCs result in a much sparser matrix of inferred connections between enzyme concentration and steady-state flux. However, these coefficients are much more interpretable as direct causality between enzyme expression and increased downstream flux. The top 10 largest and identifiable FCCs are shown in Table 2. Some pairs of enzymes and boundary fluxes, *i.e.* glycerol-3-phosphate dehydrogenase enhancing glycerol production, are direct upstream enzymes for the boundary flux in question. However, since linear pathways can have an uneven distribution of FCCs, determining the most rate-limiting step in biosynthesis pathways is an important result. Other confident FCCs represent more indirect effects: for instance, the consumption of the upstream phosphoenolpyruvate in 3-phosphoshikimate 1-carboxyvinyltransferase reduces the export of pyruvate. These results therefore show that the method, after considering experimental data, is able to suggest reasonable enzyme targets for improving the flux to a desired metabolite.

**Table 2:** Largest FCCs for the modulation of measured enzymes on measured boundary fluxes. FCC ranges represent upper and lower bounds of the 95% highest posterior density. Enzyme-boundary pairs that also appear as confident predictions prior to including experimental data are omitted.

Enzyme	Boundary	FCC Range
Glycerol 3 phosphate dehydrogenase (NAD)	Glycerol	[+0.661, +0.867]
Triose-phosphate isomerase	Glycerol	[−0.529, −0.375]
Threonine aldolase	Glycine	[+0.323, +0.420]
Pyruvate decarboxylase	Pyruvate	[−0.379, −0.308]
Pyruvate kinase	Pyruvate	[+0.207, +0.281]
Phosphofructokinase	Glycerol	[+0.150, +0.278]
ATPase cytosolic	Pyruvate	[+0.178, +0.242]
Pyruvate kinase	Ethanol	[+0.184, +0.226]
Fructose-bisphosphate aldolase	Pyruvate	[−0.244, −0.156]
3-phosphoshikimate 1-carboxyvinyltransferase	Pyruvate	[−0.219, −0.157]

## Discussion

In this study we demonstrate how kinetic models of microbial metabolism can be analyzed through modern probabilistic programming frameworks. In doing so, we have invoked approximate formalisms for enzymatic kinetics; however, we note that similar trade-offs between modeling fidelity and computational efficiency are common throughout biology and chemistry. For instance, while small-scale pathways might be better modeled at a higher level of kinetic theory, a complete kinetic description of a genome-scale kinetic model is likely currently infeasible given available data and computational resources. As biological experiments are becoming increasingly easy to iterate with modeling results, a complete kinetic description of a given pathway may not be as valuable as a reasonable guess as to how to improve a desired phenotype. Computational methodologies that quickly converge to generate a list of potential targets, such as the one proposed in this study, may therefore be essential in keeping up with the growing ease of multiomics experiments. The proposed method can also be run efficiently on consumer-grade hardware, an important factor for applications in industrial microbiology where access to large-scale high performance computing resources is limited.

As the field of variational inference is rapidly evolving, this technique could likely be made more robust or efficient through the use of alternative inference algorithms. For instance, correlations between elasticities were demonstrated through a Hamiltonian Monte Carlo trace but were missed by the corresponding mean-field Gaussian approximation. While fitting a full-rank Gaussian is likely impractical at larger data set sizes, reduced-rank approximations [37, 38] might offer a suitable compromise between posterior accuracy and computational efficiency. Additionally, inference approaches which only consider a subset of the experimental data might also prove useful. Since each perturbed state involves a new linear solve in calculating the likelihood, stochastic variational inference [39] or firefly MCMC [40] might reduce the cost of approximating or drawing samples from the posterior.

With the presented approach, genome-scale metabolic models and omics-sized datasets can be integrated to generate actionable recommendations for further strain engineering. Data at this scale has typically been analyzed through statistical “black-box” approaches that do not consider the nature of the relationship between reaction fluxes and enzyme and metabolite concentrations. Bayesian inference with linlog kinetics naturally includes stoichiometric and kinetic constraints in a scalable form. The method is also well-suited to the specific types of omics data that are currently being collected. In particular, linlog kinetics only requires relative quantification of enzyme and metabolite concentrations (rather than absolute), which are easier to collect. Additionally, the method is able to gracefully handle missing data and estimate the resulting confidence in model predictions. Despite the increasing amount of data being collected on cellular metabolism, it remains challenging to integrate this data to draw actionable conclusions. Bayesian inference with linlog kinetics is therefore broadly useful for modeling metabolism and as a tool for metabolic engineering. Future work will leverage this methodology to make predictions for subsequent experimental validation, with subsequent omics data used to iteratively refine model predictions.

## Methods

### Enabling efficient Bayesian inference through linlog kinetics

We begin with a review of the relevant equations from dynamic flux balance analysis and the linear-logarithmic kinetic framework, which together form the theoretical basis for the methodology discussed in this study. In flux balance analysis, we assume that metabolite concentrations, *x*, quickly reach a pseudo-steady state by balancing fluxes *v* through each reaction.

$$\begin{array}{c} \frac{dx}{dt}=\underset{m\times n}{N}v\left(x\right)=0, \end{array}$$ (1)

for *n* reactions and *m* metabolites, where *N*_*ij*_ indicates the stoichiometry of metabolite *i* in reaction *j*. Linlog kinetics approximates a reaction rate *v*(*x*) as a sum of logarithms [24]. Linlog kinetics is derived using the thermodynamic concept that reaction rate is proportional to the thermodynamic driving force near equilibrium [41]. Since Δ*G* = Δ*G*° + *RT* log *Q*, expressing the reaction rate as a linear combination of the logarithm of metabolite concentrations is justified. While many biochemical reactions are far from equilibrium, this relationship remains linear over a wide range of reaction affinities for enzymatic reactions [42, 43]. As an approximation, linlog kinetics does not describe enzyme-mediated kinetics as faithfully as more mechanistic frameworks [16]. However, linlog kinetics has been shown to be accurate up to 20-fold changes in metabolite concentrations [24], and for 4 to 6-fold changes in enzyme concentration relative to a reference state [25]. As a result, linlog kinetics has been used as a framework for estimating flux control coefficients (FCCs) from a range of data sources [44–47]. Most importantly, this kinetic formalism allows steady-state fluxes and metabolite concentrations as a function of enzyme expression to be determined directly via linear algebra, without the need to explicitly integrate the dynamic system until a steady-state is reached [25].

For the reaction *A* → *B* + *C*, the reaction rate is modeled as

$$\begin{array}{c} v=e(k+a\;\text{log}[A]+b\;\text{log}[B]+c\;\text{log}[C]), \end{array}$$ (2)

where *e* represents the enzyme concentration, and with coefficients *k* *a* > 0; *b*, *c* < 0. This approximation is most accurate in the vicinity of an introduced reference state, e*, v*, x* [24]. As the goal of the proposed method is to tailor enzyme expression to maximize desired fluxes, the reference state is best chosen as the current optimal performing strain. Deviations from this state can be described by the flux expression

$$\begin{array}{c} v\left(x,y\right)=\text{diag}(\frac{v^{*}e}{e^{*}})(1_{n}+\underset{n\times m}{\epsilon_{x}^{*}}\text{log}\frac{x}{x^{*}}+\underset{n\times p}{\epsilon_{y}^{*}}\text{log}\frac{y}{y^{*}}) \end{array}$$ (3)

where *x* is the concentration of intracellular (dependent) metabolite species, *y* is the concentration of *p* external (independently controllable) metabolite species, and $\epsilon_{x}^{*}$ and $\epsilon_{y}^{*}$ are sparse matrices of kinetic parameters taking the place of *a*, *b*, and *c* in Eq 2 that describe the effects of changes to metabolite concentrations on reaction rates.

In metabolic control analysis, elasticity parameters are defined that capture local effects of metabolite concentration on reaction rate [27]. These parameters are in essence a linearization of the reaction rate rule around a reference state, defined mathematically as

$$\begin{array}{c} \epsilon_{ij}^{*}\frac{x_{j}^{*}}{v_{i}^{*}}\frac{\partial v_{i}}{\partial x_{j}}. \end{array}$$

A benefit of the linlog approximation is that enzyme elasticities appear directly as kinetic parameters when normalized to a reference state (Eq 3), and therefore can intuitive values can be guessed for standard enzyme kinetics. For irreversible Michaelis-Menten kinetics for instance, the elasticity is

$$\begin{array}{ll} v & =\frac{V_{max}x}{K_{m}+x}\\ \epsilon & =\frac{x^{*}}{v^{*}}\frac{\partial v}{\partial x}=\frac{x^{*}}{K_{m}+x^{*}} \end{array}$$

Elasticities for irreversible Michaelis-Menten kinetics are therefore bound between 0 and 1, although larger elasticities are possible when reaction reversibility is considered. With an appropriately chosen elasticity, linear-logarithmic kinetics can closely approximate standard Michaelis-Menten kinetics in the vicinity of the reference state concentration, x* (S1 Fig).

Since elasticities tend to be positive for reactants, negative for products, and not be much larger than 1, reasonable starting guesses and bounds can be generated for all kinetic parameters in the model in a much easier fashion than for rate rules parameterized through traditional enzymatic expressions. Elasticities for linear-logarithmic kinetics have typically been estimated in the literature using multiple linear regression [30, 48], where estimated fluxes for each reaction are fitted as a function of their measured metabolite concentrations. However, this approach does not enforce the *Nv* = 0 constraint, nor does it allow for missing data in concentration or flux measurements. We demonstrate that incorporating steady-state constraints is computationally feasible, and that a full characterization of the posterior space can be accomplished using Hamiltonian Monte Carlo.

While linlog kinetics is a close approximation of more mechanistic rate rules, it suffers from a number of notable inconsistencies. One consequence is that fluxes can approach negative infinity as metabolite concentrations approach zero, making the framework unsuitable for describing complete pathway knockouts. However, in practice metabolite concentrations are typically expressed as log-transformed variables which cannot fall to zero. Linlog-derived rates are therefore most accurate for metabolite concentrations within one or two orders of magnitude of the reference state value. Other methodological strategies discussed later also prevent fluxes from taking unrealistic values, including using a least-norm linear solve for steady-state concentrations and clipping data to a finite range. Additionally, because it is a local approximation, the method will poorly reproduce complex enzyme dynamics at large deviations from the reference state. However, because cellular systems are constrained by homeostasis, metabolite concentrations generally do not change drastically enough to invalidate rate estimates [49]. As a result, the choice of the reference state is an important parameter in determining the effectiveness of the method. Reactions with exactly zero flux in the reference state will necessarily have zero flux in perturbed mutants. In practice, this limitation can be partially alleviated by enforcing a low absolute flux value for each reaction in the network. Since reactions can change sign in perturbed states, this allows for forward and reverse flux to be captured.

A key step in dynamic modeling of metabolic networks is solving for the steady-state concentrations and fluxes that arise from a given parameterization. Simulating this perturbation efficiently with the mathematical model is therefore a key step in estimating parameter values for the ϵ* matrices. In doing so, it is useful to define transformed variables in order to rewrite Eq 3 in a linear form (as demonstrated by Smallbone et al., 2007):

$$\begin{array}{c} \chi =\;\text{log}\;\frac{x}{x*};\;\gamma \;=\;\text{log}\;\frac{y}{y*};\;\hat{v}\;=\;\frac{v}{v*};\;\;\hat{e}\;=\;\frac{e}{e*}\\ v=\text{diag}(v*\;\hat{e})(1_{n}\;+\;\epsilon_{x}^{*}\chi \;+\;\epsilon_{y}^{*}\gamma ) \end{array}$$ (4)

Since log-transformed metabolite concentrations are linearly related to the reaction fluxes, concentrations which yield steady-state behavior can therefore be determined via a linear solve [24] after combining Eq 4 with Eq 1:

$$\begin{array}{lll} N\;\text{diag}(v*\hat{e})(1_{n}+\epsilon_{x}^{*}\chi +\epsilon_{y}^{*}\gamma ) & = & 0\\ \underset{A}{\underset{︸}{N\;\text{diag}(v*\hat{e})\epsilon_{x}^{*}\chi }} & = & \underset{b}{\underset{︸}{-N\;\text{diag}(v*\hat{e})(1_{n}+\epsilon_{y}^{*}\gamma )}}\\ \chi & = & A^{-1}b \end{array}$$ (5)

This significant result is the key advantage of linlog kinetics over alternative nonlinear rate laws. While determination of steady-state concentrations would typically require a computationally intensive ODE integration, in this approximation they can instead be calculated using a single linear solve, which are often orders of magnitude easier to compute. Additionally, it is relatively straightforward to obtain forward and reverse-mode gradients for this operation (changes in steady-state with respect to changes in kinetic parameters), a much more difficult task for ODE integration [50].

However, in general a metabolic system will contain *conserved moieties*, or metabolite quantities which can be expressed as linear combinations of other metabolites (*e.g.* ATP + ADP = constant). The stoichiometric matrix *N*, and as a result the *A* matrix defined above, will therefore not be full row rank. In effect, this means that Eq 5 has multiple solutions, each of which corresponds to a different total cofactor pool. In metabolic control theory, this problem has traditionally been solved through the introduction of a link matrix, *L*, and a reduced set of metabolites with conserved moieties removed [27, 51]. Through the link matrix, the matrix *A* can be transformed to a full-rank, square matrix and a unique steady-state can be determined that corresponds to the dynamic system’s true steady-state. However, in most biological experiments, changes to steady-state enzyme expression correspond with separately cultured cell lines for which the assumption that total cofactor pools would remain constant is not necessarily valid. Instead, we propose that a more biologically relevant solution to Eq 5 is one that minimizes ‖*χ*‖_2_: *i.e.,* the solution that results in the smallest deviation of metabolite concentrations from the reference state. This assumption has experimental support in that intracellular metabolite concentrations tend to be buffered from drastic changes through feedback circuits at the genetic and enzyme level [49]. We therefore calculate steady-state metabolite concentrations through a pseudoinverse,

$$\begin{array}{c} \chi_{ss}=A^{†}b \end{array}$$ (6)

A derivation of the forward and reverse-mode gradients for the regularized linear solve operation is included in S1 Text. We note that in practice, numerical stability is improved if *A* can be made full row-rank prior to the least-norm linear solve. We can therefore replace *N* with $\tilde{N}$ (by removing rows corresponding to redundant conservation relations) in order to form a wide *A* matrix (with more columns than rows) prior to performing the least-norm linear solve in Eq 6. Since a flux vector that satisfies $\tilde{N}\;v=0$ will also satisfy *N v* = 0, this change can be made without affecting the final solution.

Due to the changes to traditional MCA theory introduced by the altered steady-state calculation defined above, we also slightly modify the traditional calculations of metabolite and flux control coefficients.

$$\begin{array}{llll} C_{x,kj}^{*} & = & \frac{e_{j}^{*}}{x_{k}^{*}}\frac{dx_{k}}{de_{j}} & =-{\left(\tilde{N}\;\text{diag}\left(v^{*}\right)\epsilon_{x}^{*}\right)}^{†}\tilde{N}\;\text{diag}\;v^{*}\\ C_{v,ij}^{*} & = & \frac{e_{j}^{*}}{v_{i}^{*}}\frac{dv_{i}}{de_{j}} & =\epsilon_{x}^{*}C_{x}^{*}+I \end{array}$$

Since flux and metabolite control coefficient matrices describe the response of the steady-state to changes in enzyme expression, our altered versions describe the flux response at the particular steady-state in which metabolite concentrations are as close as possible to the unperturbed state. In practice, this has the effect of improving the identifiability of FCCs in the numerical experiments described below. A plot of FCC values obtained via both traditional and modified methods for the following genome-scale model is shown in S10 Fig, indicating that either both methods tend to yield a similar result, or that the identifiability of the link-matrix FCC is particularly poor, with the pseudoinverse FCC pulled close to zero.

In Bayesian inference, a *likelihood* model, *p*(*z*|*θ*), is constructed for the probability of observing the measured data, *z*, given particular values for each parameter, *θ*. Combined with a *prior* distribution, *p*(*θ*), for each parameter that represents generally feasible values, numerical approaches use Bayes theorem,

p(θ|z)∝p(z|θ)p(θ)

to estimate the *posterior* parameter distribution *p*(*θ*|*z*): the probability a parameter takes the given value after accounting for the observed data. With a suitable kinetic framework for calculating steady-state fluxes and concentrations as a function of enzyme expression, we next discuss the prior distributions and likelihood function required for Bayesian inference. The prior distributions represent our belief of possible parameter values before any experimental data is collected. For metabolite elasticity matrices we assume that for any given reaction, reactants are likely to be associated with a positive elasticity, while products likely have a negative elasticity (increasing reactant concentration increases reaction rate, while increasing product concentration decreases reaction rate). Alternatively, we assume that if a metabolite does not directly participate in a reaction, it can only regulate the reaction if it appears in the same sub-cellular compartment. We denote the vectors *c*_*m*_ and *c*_*r*_ of metabolite and reaction compartments, respectively. Since regulation of enzymatic reactions by otherwise nonparticipatory metabolites is relatively rare, we place a sparsity-inducing prior on its elasticity value. This distribution encourages elasticities for off-target metabolites to take values near zero, unless strong experimental evidence for a regulator interaction is present. The combined priors for enzyme elasticities can then be expressed through the following functional form, also depicted graphically in Fig 1.

$$\begin{array}{c} \epsilon_{x,ji}^{*}∼\{\begin{array}{cc} \mathrm{sign}\left(-N_{ij}\right)\cdot \text{HalfNormal}\left(\sigma =1\right) & \text{if}\;N_{ij}\neq 0\\ \text{Laplace}(\mu =0,b=0.05) & \text{if}\;N_{ij}=0\;\text{and}\;c_{m,i}=c_{r,j}\\ 0 & \text{if}\;N_{ij}=0\;\text{and}\;c_{m,i}\neq c_{r,j} \end{array} \end{array}$$ (7)

We note that the assumption that reactants and products must take positive and negative elasticity values, respectively, can be relaxed by replacing the half-normal distribution in Eq 7 with a skew-normal distribution with a positive shape parameter. This distribution reflects the belief that while reactants typically take positive elasticities, rare cases may exist where substrate inhibition results in a negative slope of reaction rate with respect to substrate concentration. While substrate inhibition has been shown to be of biological importance [52], in practice this choice of a prior distribution results in less robust convergence to a stable posterior distribution and was avoided in higher-dimensional inference problems.

An explicit likelihood function can be formed by constructing a statistical model for the observed data. We assume that observed data are normally distributed around the calculated steady-state metabolite and flux values.

$$\begin{array}{lll} \chi_{obs} & \sim & \text{Normal}(\chi ,\;\sigma_{x}^{2})\\ {\hat{v}}_{obs} & \sim & \text{Normal}(\hat{v},\;\sigma_{v}^{2}) \end{array}$$ (8)

Experimental errors, *σ*_*x*_ and *σ*_*v*_, can either be set explicitly or estimated from the data. For smaller-scale examples, we place half-normal priors on these variables, while for larger datasets we set these values explicitly to improve numerical stability. We also note that for genome-scale multiomics data, computational stability can be improved by fitting log-transformed normalized fluxes,

$$\text{log}\;{\hat{v}}_{obs}∼\text{Normal}\left(\text{log}\;\hat{v},\sigma_{v}^{2}\right),$$

so that flux, metabolite, and enzyme expression data fall on similar orders of magnitude. While this assumption comes at the cost of preventing measured fluxes from reversing directions between perturbed states, this restriction was not significant for the examples considered in this study. However, this framework could be easily extended to handle situations where a measured flux reverses directions between experimental conditions. Most simply, the reversible reaction could be withheld from the log transform and fit in linear space. Alternatively, if separate estimates for the forward and reverse flux could be obtained, as is often the case in ^13^C labeling studies, the reaction could be decomposed and modeled separately as irreversible forward and reverse reactions.

Once the prior distribution and likelihood model have been specified, the remaining task is to numerically estimate posterior distributions in elasticity parameters. Two inference algorithms were used, the No-U-turn sampler (NUTS) and Automatic differentiation variational inference (ADVI). NUTS [28], as a variant of Hamiltonian Monte Carlo (HMC), constructs an iterative process (a Markov chain) that eventually converges to the true posterior distribution. Markov chain Monte Carlo methods, while accurate, are computationally intensive and likely limited in application to smaller-scale models and datasets. While the major computational bottleneck in metabolic ensemble modeling (integrating an ODE until steady state) has been removed, calculating the likelihood function still involves a separate linear solve for each steady-state experimental condition. Therefore as model sizes approach the genome-scale, HMC methods quickly become computationally infeasible. Variational methods, however, offer an alternative to Markov chain Monte Carlo methods that can scale to models with thousands of parameters. ADVI approximates the posterior distribution by a simple, closed-form probability (typically Gaussian), then estimates parameters for the approximate posterior to minimize the distance between the true and approximated distribution [29].

### Characterization of an *in vitro* linear pathway

Since all metabolites (including external species) are present in the same compartment, all elasticities are allowed to have allosteric interactions normalized with Laplace priors. Measurement errors in fluxes and metabolite concentrations were fit by the inference algorithm by placing a half-normal prior distribution on the *σ* values in Eq 8. The same reference steady-state was chosen (experiment 2) as was done by Wu et al., 2004. Prior distributions in enzyme elasticities were parameterized in a similar fashion to Eq 7, except that a SkewNormal(*σ* = 1, *α* = 5) distribution was used in place of the HalfNormal when *N*_*ij*_ ≠ 0. This distribution allows a small chance of substrate inhibition, where higher concentrations of a reactant in an enzyme-catalyzed reactions actually reduces reaction rate. However, this distribution was not used in larger examples as it introduced additional numerical instability. A comparison of the HalfNormal, SkewNormal, and Laplace distributions (with parameters used in this work) is shown in S2 Fig.

Using NUTS, stable traces were found across four independent chains, indicating that each trace converged to the true posterior distribution (Fig 2B, S3 Fig). For this small-scale example, NUTS took less than 10 minutes on a single computer. Applying ADVI to this example, the evidence lower bound (ELBO), a measure of the closeness of fit between the approximated and true posterior distribution, converged after approximately 10,000 iterations of stochastic gradient descent (S4 Fig). A full 25,000 iterations were completed in under 40 seconds on a single computer. Resulting posterior distributions were deemed confidently nonzero if the 95% highest posterior density (HPD) interval did not overlap zero.

Comparing the mean and variance of the elasticity posterior distributions from the two different approaches, we notice that while the mean values agree closely, ADVI underestimates the variance for many parameters (S5 Fig). This underestimation is typical of mean-field ADVI [29], and might be alleviated in the future through more advanced variational methods [37].

### Determining optimal enzyme targets from limited data

As the goal of the inference approach is to estimate targets for subsequent lysine flux improvement, we chose the reference state for linlog kinetics to be the final, optimized strain with 5 overexpressed enzymes. Since the reference state was chosen to be the final, optimized strain, perturbed strains had lower relative enzyme concentrations and lysine flux. Values for target lysine fluxes were taken from the published yields for the W3110 strain, with a calculated glucose uptake flux of 1.243 mol L^−1^week^−1^. As the resulting enzyme concentrations following overexpression were unknown, prior distributions were placed on these parameters. Since enzyme concentrations were specified relative to the overexpressed reference state, uniform priors between zero and one were placed on the relative concentrations of each of the 5 modulated enzymes. The resulting inferred distributions in enzyme expression for the 5 enzymes are shown in S6 Fig.

When analyzed with metabolic ensemble modeling, each successive enzyme overexpression was required to increase lysine flux over the previous base strain. However in our framework, we require a continuous and differentiable likelihood function, and are also able to directly incorporate the experimentally measured yields. Prior distributions in enzyme elasticities were specified as described in Eq 7, and since the dataset did not include changes in external metabolites, no *ϵ*_*y*_ values were needed. Prior distributions in elasticities associated with stoichiometric metabolite-reaction pairs (kinetic elasticities) used a half-normal distribution with *σ* = 1 and had a 95% HPD that spanned from 0 to 2. Kinetic elasticities were therefore flagged as constrained by the data if they had a 95% highest posterior density that spanned less than 0.75 elasticity units. Regulatory elasticities and FCCs were deemed confidently nonzero if their 95% HPD did not overlap zero.

### Informing strain design through multiomics

As the goal in this example is to demonstrate that linlog kinetics are able to consume large amounts of multiomics data, a reference state near the center of the considered data was chosen, specifically the chemostat with phosphate-limiting media at a 0.11 hr^−1^ dilution rate. Reference fluxes (v*) were calculated via flux balance analysis by minimizing error with the experimental boundary measurements while enforcing a nonzero flux through each reaction. In this example, relative metabolite concentrations are given as log2-transformed values [53]. Even with an unknown pre-exponential constant *A*, relative concentrations *χ* can be calculated from log2-transformed concentrations *a* and *b*:

$$\chi =\text{log}(\frac{x}{x*})=\text{log}(\frac{A2^{a}}{A2^{b}})=\left(a-b\right)\text{log}\;2.$$

Distributions of the transformed data are shown in Fig 4A, indicating that the majority of data falls within one order of magnitude from the reference state value (values shown are natural logs). Priors in enzyme elasticities were specified as in Eq 7, except that Laplace distributions for off-target regulations (*N*_*ij*_ = 0) were given a *b* = 0.01, which was found to have an optimal trade-off between regularization and flexibility for the larger model. The effect of this regularization for several values of *b* was explored, as shown in S7 Fig. The ability of the model to fit the experimental data and the number of confidently identified nonzero regulatory elasticities were relatively consistent for values of *b* from 0.01 to 0.1.

Allowing all enzyme concentrations to vary induces a trade-off where steady-state fluxes are controlled through changes to enzyme expression instead of changes to steady-state metabolite concentrations. While Hackett *et al*., 2016 have previously shown that metabolic control is mainly determined by metabolite concentrations, some mechanism for adjusting enzyme levels is required to buffer against errors in model formulation and experimental measurements. By placing a Laplace prior on unmeasured enzymes, we create a regularizing effect that penalizes an over-reliance on enzymatic control:

$$\begin{array}{c} \text{log}\;{\hat{e}}_{i}∼\{\begin{array}{cc} \text{Normal}(\mu =\text{log}\left({\hat{e}}_{i,obs},\sigma =0.2\right) & \text{if}\;e_{i}\;\text{measured}\\ \text{Laplace}(\mu =0,b=0.1) & \text{if}\;e_{i}\;\text{unmeasured}\\ 0 & \text{if}\;\text{reaction}\;i\;\text{uncatalyzed} \end{array} \end{array}$$

The model also has to consider changes in the external metabolite concentrations between media formulations and dilution rates. We therefore place vague priors on the external concentrations of imported substrates, including glucose, phosphate, sulfate, nitrogen, and oxygen:

$$\begin{array}{c} \gamma ∼\text{Normal}(\mu =0,\sigma =10). \end{array}$$

Observed steady-state metabolite concentrations and fluxes are incorporated through a likelihood model that assumes experimental error is normally distributed around log-transformed metabolite and boundary flux data. Standard deviations were chosen as *σ*_*x*_ = 0.2 for the metabolite data and *σ*_*v*_ = 0.1 for the log-transformed fluxes. To improve numerical stability, we also clip the log-transformed, relative experimental data to ±1.5, such that log-transformed experimental data and model predictions greater than 1.5 or less than -1.5 are replaced by ±1.5. This process has the effect of reducing the influence of extreme points, especially in regimes far from the reference state that are unlikely to be fit well by the linlog approximation. However, the model is still required to predict the directionality and high-magnitude of these points correctly. Fitting the model using ADVI required 40,000 iterations of stochastic gradient descent, taking approximately five hours on a single compute node (S8 Fig). Median absolute errors between the model predictions (median of the posterior predictive distribution) and experimental data points are 0.124, 0.0952, and 0.0186 for log-transformed metabolite, flux, and enzymes, respectively, for normalized points that fall within the [−1.5, 1.5] window. Similarly to previous examples, FCCs and elasticities are determined to be confidently nonzero if they have a 95% HPD that crosses zero.

### Software availability

All simulations were performed in Python using the pymc3 library [54]. Additional code to initialize the elasticity prior matrices and calculate the steady-state metabolites and fluxes is provided at github.com/pstjohn/emll, along with jupyter notebooks detailing the use cases described above. Classes are defined to perform the steady-state calculations in both numpy and Theano. For steady-state calculations in Theano, gradients must also be provided that allow the inference algorithms to simultaneously calculate the derivative of the likelihood result with respect to estimated parameter values. This gradient calculation is provided as a Theano operation, with the mathematical derivation for these reverse-mode gradients provided in S1 Text. These calculations interface directly with the underlying LAPACK routines for the regularized linear solve. Probability models are therefore constructed as detailed in the pymc3 documentation, using the additional routines provided to expedite construction of prior distributions for the elasticity variables and performing the steady-state calculation of fluxes and metabolite concentrations.

## Supporting information

S1 Fig Linear-logarithmic kinetics. The rate law for linlog kinetics closely approximates Michaelis-Menten kinetics in the vicinity of the reference state.
(EPS)

S2 Fig Distributions used to specify priors on enzyme elasticities. The HalfNormal distribution is used when a metabolite directly participates in a reaction, and the directionality is known. The SkewNormal distribution is similar to the HalfNormal, but also allows for the small possibility of substrate inhibition. Laplace distributions were used to specify potential regulatory interactions with an unknown direction.
(EPS)

S3 Fig NUTS trace for the *in vitro* dataset. (left) kernel density estimates of each parameter. Vertical bars indicate the values obtained using the multiple linear regression technique of Wu *et al*. [30]. (right) Samples from the MCMC sampler.
(EPS)

S4 Fig Convergence of the Evidence Lower Bound (ELBO) for the *in vitro* dataset. (EPS)

S5 Fig NUTS vs ADVI estimation. Comparison between posterior distributions for the *in vitro* dataset as estimated by NUTS (solid lines) or ADVI (dashed lines). ADVI posteriors have a similar mean but smaller variance.
(EPS)

S6 Fig Posterior distributions in enzyme expression prior to exogenous amplification. (EPS)

S7 Fig Effect of regularization for the yeast model. (A) Posterior predictive distributions as a function of the *b* parameter is the Laplace prior for enzyme elasticities. The ability of the model to reproduce the data is quantified through the use of the median absolute error (MAE). (B) Percent of confidently inferred regulatory (off-target) elasticities. Elasticities were deemed confidently inferred if their 95% HPD did not include zero.
(EPS)

S8 Fig Convergence of the Evidence Lower Bound (ELBO) for the multiomics dataset and yeast metabolic model. (EPS)

S9 Fig Full heatmaps (with labeled boundary fluxes and enzymes) for (A) correlation coefficients and (B) FCCs estimated via the multiomics dataset. Full names for the reaction IDs shown can be found in the detailed model description.
(EPS)

S10 Fig Comparison of FCCs calculated with the link matrix approach and with the pseudoinverse. (EPS)

S1 Text Calculating reverse-mode gradients for regularized linear solve. (PDF)
