# Supplementary material for: Bayesian inference of metabolic kinetics from genome-scale multiomics data
Source: PLoS Comput Biol. 2019 Nov 4;15(11):e1007424. doi: 10.1371/journal.pcbi.1007424 (PMC6855570; doi:10.1371/journal.pcbi.1007424)
Supplement: S1 Text — (PDF) [file pcbi.1007424.s011.pdf]

Bayesian inference of metabolic kinetics from genome-scale multiomics data

Peter C. St. John[1], Jonathan Strutz[2], Linda J. Broadbelt[2], Keith E.J. Tyo[2], Yannick J. Bomble[1,*]

[1]Biosciences Center, National Renewable Energy Laboratory, 15013 Denver West Parkway, Golden CO 80401, USA
[2]Department of Chemical and Biological Engineering, Northwestern University, 2145 Sheridan Road, Evanston IL 60208, USA
[*]Email: Yannick.Bomble@nrel.gov

Calculating reverse-mode gradients for regularized linear solve

In order to efficiently perform many inference approaches, forward and reverse-mode gradients for the likelihood function are required. In this method, the least-squares linear solve is a particularly tricky operation for which gradients in some automatic differentiation packages are not automatically supplied. In this section, we therefore derive the necessary matrix equations to calculate forward and reverse mode gradients for the least-norm linear solve, $\chi_{ss} = \mathbf{A}^{+}\mathbf{b}$. In practice, it is much more efficient to calculate this least-norm solution directly (*i.e.*, using the LAPACK routine `dgelsy`) instead of explicitly calculating the pseudoinverse matrix.

Gradients for the least-norm solution are derived by first calculating those for Tikhonov regularization, and subsequently taking the limit as $\lambda \to 0$. Definitions for matrix derivatives are taken from (1). Similar to example 2.3.1 in Giles (2008), the forward derivative for a Tikhonov-regularized linear takes the form

$$C = (\underbrace{A^T A + \lambda I}_{D})^{-1} \underbrace{A^T B}_{E}$$

$$\begin{aligned}
dC &= D^{-1}(dE - dD\, C) \quad \text{from } C = D^{-1}E \\
dD &= dA^T A + A^T dA \\
dE &= dA^T B + A^T dB
\end{aligned}$$

Substituting into the equation for dC,

$$\begin{aligned}
dC &= (A^T A + \lambda I)^{-1} \left(dA^T B + A^T dB - (dA^T A + A^T dA) C \right) \\
dC &= (A^T A)^{-1} \left(dA^T B + A^T dB - (dA^T A + A^T dA) C \right)
\end{aligned}$$

Also following Giles (2.3.1), the reverse mode gradient can be found:

$$\begin{aligned}
\mathrm{Tr}(\bar{C}^T dC) &= \mathrm{Tr}(\bar{C}^T D^{-1} dE) - \mathrm{Tr}(\bar{C}^T D^{-1} dD\, C) \\
&= \mathrm{Tr}(\bar{C}^T D^{-1} dA^T B) + \mathrm{Tr}(\bar{C}^T D^{-1} A^T dB) \\
&\quad - \mathrm{Tr}(\bar{C}^T D^{-1} dA^T A\, C) - \mathrm{Tr}(\bar{C}^T D^{-1} A^T dA\, C) \\
&= \mathrm{Tr}\left((B - AC)\bar{C}^T D^{-1} dA^T \right) - \mathrm{Tr}(C\bar{C}^T D^{-1} A^T dA) \\
&\quad - \mathrm{Tr}(\bar{C}^T D^{-1} A^T dB) \\
&= \mathrm{Tr}\left[\left(D^{-T} \bar{C} (B - AC)^T - C\bar{C}^T D^{-1} A^T \right) dA \right] \\
&\quad - \mathrm{Tr}(\bar{C}^T D^{-1} A^T dB)
\end{aligned}$$

therefore,

$$\begin{aligned}
\bar{B} &= \left(\bar{C}^T D^{-1} A^T\right)^T = A D^{-T} \bar{C} \\
\bar{A} &= \left(D^{-T}\bar{C}(B - AC)^T - C\bar{C}^T D^{-1} A^T\right)^T \\
&= (B - AC)\bar{C}^T D^{-1} - \underbrace{A D^{-T} \bar{C}}_{\bar{B}} C^T \\
&= (B - AC)\bar{C}^T D^{-1} - \bar{B} C^T
\end{aligned}$$

Since $D = A^T A + \lambda I = (A^T A + \lambda I)^T$, these gradients can be further simplified using the relations

$$\begin{aligned}
Dx &= \bar{C} \\
x &= D^{-1}\bar{C} \\
x^T &= \bar{C}^T D^{-1}
\end{aligned}$$

After substituting into the equations for $\bar{A}$ and $\bar{B}$, we are left with

$$\begin{aligned}
\bar{B} &= Ax \\
\bar{A} &= (B - AC)x^T - \bar{B} C^T
\end{aligned}$$

as the reverse-mode gradients for the least-norm solve $C = A^{\dagger} B$.

References

1. Giles MB. Collected matrix derivative results for forward and reverse mode algorithmic differentiation. Advances in Automatic Differentiation. 2008;35–44.
